# We need to talk about data! Transforming the conversation between researchers and the public

**DOI:** 10.23889/ijpds.v8i2.2325

**Published:** 2023-09-14

**Authors:** Richard Welpton

**Affiliations:** 1 UKRI, United Kingdom

## Objectives

Our Future Data Services (FDS) work has shown that researchers find it difficult to start conversations with the public about their work and the use of data. This session will explore how data services could effectively facilitate conversations between the public and researchers in the future.

## Methods

We know that the public is much more aware that data about them are collected: sometimes through mistakes made, leading to public engagement as an afterthought.

Researchers have told ESRC that they would like to engage with the public; but barriers prevent them from doing so. ESRC would like to run this workshop to find out from researchers what barriers they believe prevent their engagement with the public.

We’ll facilitate face to face discussions as a deep dive to understand what these barriers are, and what resources would help researchers to meaningfully connect with the public in the future.

## Results

The input collected from researchers at this workshop will help us draft recommendations about how ESRC/UKRI funded data services can in the future support researchers to engage with the public when designing research proposals that use personal data.

These recommendations will likely identify specific funding opportunities that ESRC can target to enable data services to support researchers to engage with the public. For example, resources could be provided through data service website, such as training materials (including crib sheets and videos) and signposting to organisations that can enable them to speak to the public about how they would like to use their data for research.

These recommendations will be published in Spring 2024 and funding opportunities could be made available from 2025 onwards.

## Conclusion

ESRC has heard researchers say they would like to work with the public when designing and undertaking research with personal data in the future. But routine engagement with the public is a hard ask; unless supported through specific programmes, navigating the public engagement remains niche, yet essential.

